# Differentiated cytoplasmic granule formation in quiescent and non-quiescent cells upon chronological aging

**DOI:** 10.15698/mic2016.03.484

**Published:** 2016-03-03

**Authors:** Hsin-Yi Lee, Kuo-Yu Cheng, Jung-Chi Chao, Jun-Yi Leu

**Affiliations:** 1 Molecular and Cell Biology, Taiwan International Graduate Program, Graduate Institute of Life Sciences, National Defense Medical Center and Academia Sinica, Taipei, Taiwan.; 2 Department of Life Sciences and Institute of Genome Sciences, National Yang-Ming University, Taipei, Taiwan.; 3 Institute of Molecular Biology, Academia Sinica, Taipei, Taiwan.

**Keywords:** stationary phase, chronological aging, quiescent cells, cytoplasmic granules, Hsp42

## Abstract

Stationary phase cultures represent a complicated cell population comprising at least two different cell types, quiescent (Q) and non-quiescent (NQ) cells. Q and NQ cells have different lifespans and cell physiologies. However, less is known about the organization of cytosolic protein structures in these two cell types. In this study, we examined Q and NQ cells for the formation of several stationary phase-prevalent granule structures including actin bodies, proteasome storage granules, stress granules, P-bodies, the compartment for unconventional protein secretion (CUPS), and Hsp42-associated stationary phase granules (Hsp42-SPGs). Most of these structures preferentially form in NQ cells, except for Hsp42-SPGs, which are enriched in Q cells. When nutrients are provided, NQ cells enter mitosis less efficiently than Q cells, likely due to the time requirement for reorganizing some granule structures. We observed that heat shock-induced misfolded proteins often colocalize to Hsp42-SPGs, and Q cells clear these protein aggregates more efficiently, suggesting that Hsp42-SPGs may play an important role in the stress resistance of Q cells. Finally, we show that the cell fate of NQ cells is largely irreversible even if they are allowed to reenter mitosis. Our results reveal that the formation of different granule structures may represent the early stage of cell type differentiation in yeast stationary phase cultures.

## INTRODUCTION

Studies in the budding yeast *Saccharomyces cerevisiae* have contributed considerably to our knowledge of aging-related genes and pathways 1. In yeast, two distinct models of aging processes have been established: replicative aging and chronological aging. The model of replicative aging defines lifespan by the number of daughter cells that a mother cell can produce before senescence 2. The chronological lifespan (CLS) is defined by the time that a yeast cell can survive in a non-dividing state in stationary phase cultures 3.

In rich medium containing glucose, yeast cells proliferate logarithmically using energy generated from glucose fermentation rather than respiration. When glucose supplies become limiting, in order to use available non-fermentable carbon sources yeast cells enter diauxic shift that changes cell metabolism from fermentation to respiration. After all carbon sources are exhausted, cells will eventually enter the stationary phase 3. The CLS is measured by monitoring the ability of stationary phase cells to reenter mitotic growth over time when fresh carbon sources are provided. Thus, understanding the physiological factors that influence cell-cycle reentry of stationary phase cells can provide insights into the mechanism of CLS.

Previous studies have observed that non-proliferating stationary phase cells exhibit several specific features; they accumulate glycogen and trehalose, develop thickened cell walls 4, and become more resistant to thermo- and osmo-stress compared to log-phase cells 5. In addition, both transcription and protein synthesis are reduced 6,7, and autophagy is induced 8. Stationary phase cells also display specific gene expression profiles. For example, the ribosomal genes are repressed and a subset of genes, including stress response genes such as *HSP26* and *HSP42*, are strongly induced 4,9. These characteristics are thought to play roles in the maintenance of cell viability during the stationary phase.

Yeast cells in stationary phase cultures are not homogeneous. Two different cell types, quiescent (Q) and non-quiescent (NQ) cells, can be separated from yeast stationary phase cultures using the Percoll density gradient 10. Q cells are more resistant to stress, exhibit a high respiratory rate, and stay reproductively competent for a longer period of time. In contrast, NQ cells are sensitive to heat shock and lose their reproductive ability quickly 10,11. Examination of soluble mRNAs in Q cells has revealed enrichment of genes related to vesicle transport, oxygen and ROS metabolism, membrane organization, lipid metabolism and signal transduction, which may be responsible for their long-term survival under starvation. In contrast, NQ cells have been found to express genes related to Ty element transposition, and DNA recombination and metabolism, which are relevant to the high mutability of NQ cells 9. Consistent with these mRNA expression profiles, the abundance of individual proteins can also be very different between Q and NQ cells 11. Thus, Q and NQ cells are physiologically distinct populations in stationary phase cultures and this fact could potentially complicate studies of the CLS model in yeast. Some observed stationary phase-specific features may only exist in Q or NQ cells, but not in both.

Recently, cytosolic protein granule formation has been found to be a widespread phenomenon in stationary phase cells 12. A systematic screen of about 800 cytosolic proteins revealed that 180 of them formed cytosolic punctate foci in stationary phase cells. Some of the punctuate foci were shown to be triggered by the absence of specific nutrients 12. In addition, actin and proteasomes are reported to translocate and form cytosolic granule structures, which are hypothesized to serve as a protein reservoir in stationary phase cells 13,14. Ribonucleoprotein granules such as the processing body (P-body) and stress granules are formed prior to and after stationary phase arrest, respectively 15. Furthermore, the formation of P-bodies is probably involved in the maintenance of cell viability in the stationary phase 15,16. Upon starvation, some Golgi-related proteins are relocated to a granule-like compartment called the compartment for unconventional protein secretion (CUPS). CUPS is suggested to sort and secrete proteins that do not enter the regular ER-Golgi secretion pathway 17,18. During stationary phase, the small heat shock protein Hsp42 also forms granules that contain many other components, including a histone deacetylase Hos2 19. The Hsp42-associated granules probably execute multiple functions in stationary phase cells. In general, the abovementioned protein granules are reversible structures and have been suggested to function as storage or act as quality control of proteins and RNA. Nevertheless, it remains unclear how different granules are organized in stationary phase cells and whether the formation of different types of granules represents distinct physiological states of stationary phase cells.

In this study, we examined the organization of different cytosolic protein granules and the cell physiology of Q and NQ cells. Interestingly, most granule structures including actin bodies, proteasome storage granules (PSG), stress granules, and P-bodies were enriched in NQ cells. Only Hsp42-associated stationary phase granules (Hsp42-SPGs) preferentially formed and were enhanced in Q cells. When nutrients were provided, Q cells were able to quickly re-enter the cell cycle, but NQ cells needed more time to reorganize the actin bodies before exiting stationary phase. Finally, we show that the cell fate of NQ cells was mainly deterministic. Even if NQ cells were induced to re-enter mitosis for several cell cycles, most of them still differentiated into NQ cells upon nutrient starvation.

## RESULTS

### Most granule structures are enriched in non-quiescent cells in stationary phase cultures.

Previous studies have shown that stationary phase cell cultures comprise Q and NQ cells that exhibit different cell physiologies 9,10. In addition, several granule-like structures have been observed in stationary phase cultures 13,14,15,18,19. However, it remains unclear how different granule structures are organized in these two types of cells. To address this question, we selected at least two protein components from each granule structure that had been tagged with GFP and examined their distributions in Q and NQ cells (Table 1). Yeast strains carrying these GFP-fusion genes were grown in YPD for 5 days and then fractionated into Q and NQ cells (see Materials and Methods) 10.

**Table 1:** Formation frequencies of stationary phase-specific granules in Q and NQ cells. Percentages of cells displaying granule structures are shown. The representative images of granule-containing cells of different strains are shown in Figures 1 and S2. At least 3 samples were analyzed for each strain and at least 100 cells were counted in each sample. S.E.: standard errors. p values were calculated using the two-tailed Student’s t-test. *: p < 0.05; **: p < 0.01; ***: p < 0.005.

		**NQ cells**			**Q cells**		
		Mean [%]	SE [%]		Mean [%]	SE [%]		p value
P-body	Edc3	76	1.2		37	3.7		***
	Dcp2	84	2.9		72	2.5		**
	Pat1	60	4.5		44	3.1		*
	Pby1	86	2.2		45	2.1		***
Actin body	Abp1	85	2.9		2	1.5		***
	Cap1	92	2.2		5	1.2		***
	Cap2	76	2.2		5	2.6		***
	Abp140	77	5.2		3	0.6		***
	Crn1	74	4.7		3	1.3		***
Stress granule	Ygr250c	33	4.1		0	0		***
	Cdc33	54	5.5		1	0.3		***
CUPS	Grh1	78	1.5		73	3.2		0.205
	Uso1	48	5.9		49	4.7		0.836
PSG	Pre6	82	1.3		12	1.5		***
	Pre2	39	2.3		2	0.9		***
	Pre3	69	3.8		36	3.2		***
	Pup1	86	1.8		35	1.5		***
	Rpn1	61	4.9		37	0.6		***
Hsp42-SPG	Hsp42	80	1.0		88	3.7		*
	Hsp26	35	1.2		85	2.4		***

**Figure 1:**
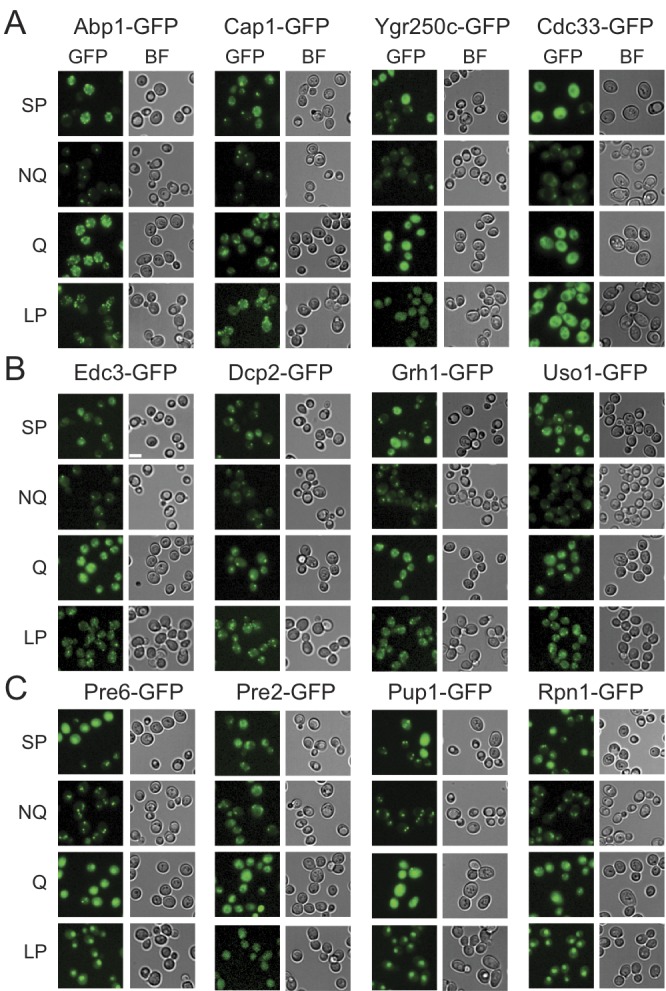
FIGURE 1: Differential formation of stationary phase-specific granules in Q and NQ cells. Yeast strains carrying the GFP-tagged genes were grown in YPD for 5 days to enter stationary phase and then cells were fractionated using the Percoll gradient to isolate the Q (in the lower layer) and NQ cells (in the upper layer). The fluorescence images of total stationary phase cultures (SP) and the fractionated cells (Q and NQ) from the same strain are shown under identical brightness and contrast settings. Log-phase (LP) images were taken from exponential cell cultures whose OD_600_ values were between 0.4 and 0.6. Scale bar: 5 μm. BF: brightfield. **(A)** Components of the actin body (Abp1 and Cap1) and stress granule (Ygr250c and Cdc33). **(B)** Components of the P-body (Edc3 and Dcp2) and CUPS (Grh1 and Uso1). **(C)** Components of the proteasome storage granule (Pre6, Pre2, Pup1 and Rpn1). In most strains, the GFP intensity of granule marker proteins appears to be stronger in Q cells, which is probably due to the fact that the genes encoding these marker proteins have a higher mRNA abundance in Q cells 9.

Microscopic examination of the fractionated cells revealed that the granule structures indeed showed very distinct organizations in Q and NQ cells (Table 1). Components of actin bodies (Abp1, Abp140, Cap1, Cap2 and Crn1) and stress granules (Ygr250c and Cdc33) almost exclusively formed granules in NQ cells. In Q cells, these proteins displayed localization patterns similar to those in log-phase cells (Figure 1A and S1). Components of P-bodies (Dcp2, Edc3, Pat1 and Pby1) and PSGs (Pre2, Pre3, Pre6, Pup1 and Rpn1) formed granules in both types of cells, but with significantly higher frequencies in NQ cells. In addition, when PSGs formed in Q cells, they exhibited a different pattern from that in NQ cells. Cytosolic PSGs in Q cells were usually accompanied by strong fluorescence intensities in the nucleus, which is the original localization of proteasomes in log-phase cells (Figure 1B and 1C). CUPS is the only structure whose components (Grh1 and Uso1) formed granules in both Q and NQ cells with similar frequencies and intensities (Figure 1B).

**Figure 2:**
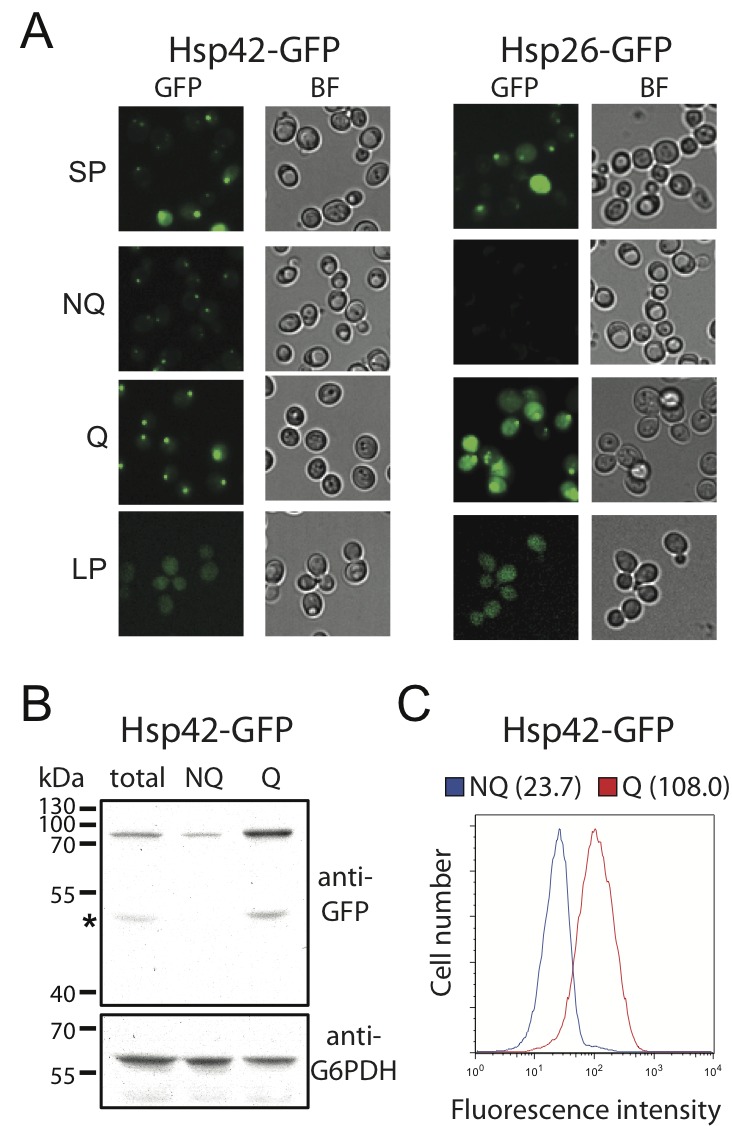
FIGURE 2: The formation of Hsp42-SPGs is enhanced in Q cells. **(A)** Images of stationary phase, log phase, Q and NQ cells carrying GFP-tagged Hsp42 or Hsp26. The fluorescence intensity of Hsp42-SPGs in Q cells is generally brighter than that in NQ cells. Also see Fig. 1 for the detailed experimental description. **(B)** Western blot results show that Hsp42-GFP is up-regulated in Q cells. Protein from total stationary phase, Q, and NQ cells was extracted and detected using anti-GFP and anti-G6PDH antibodies. The signal intensity of the Western blot was quantified. The normalized mean values and standard errors of relative ratios of Hsp42-GFP/G6PDH from three biological repeats are 1.0 ± 0.11, 0.5 ± 0.10, and 3.3 ± 0.21 for total stationary phase cultures, NQ cells and Q cells, respectively. The asterisk indicates the degradation form of Hsp42-GFP. **(C)** Flow cytometry results show that the fluorescence intensity of Hsp42-GFP in Q cells is higher than that in NQ cells. Numbers in the parentheses are the median Hsp42-GFP intensities of Q and NQ cells.

Unlike other granule structures, Hsp42-SPGs were more likely to be found in Q cells (Table 1). Moreover, the fluorescence intensity and granule size of Hsp42-SPGs in Q cells were visibly greater than that in NQ cells (Figure 2A). Flow cytometry and Western blotting revealed that the protein abundance of Hsp42 is significantly higher in Q cells compared to NQ cells (Figure 2B and 2C). These results suggest that Hsp42-SPGs are intrinsically different from the other granule structures and may play a specific role in Q cells.

### Heat shock-induced misfolded proteins colocalize with Hsp42-stationary phase granules.

**Figure 3:**
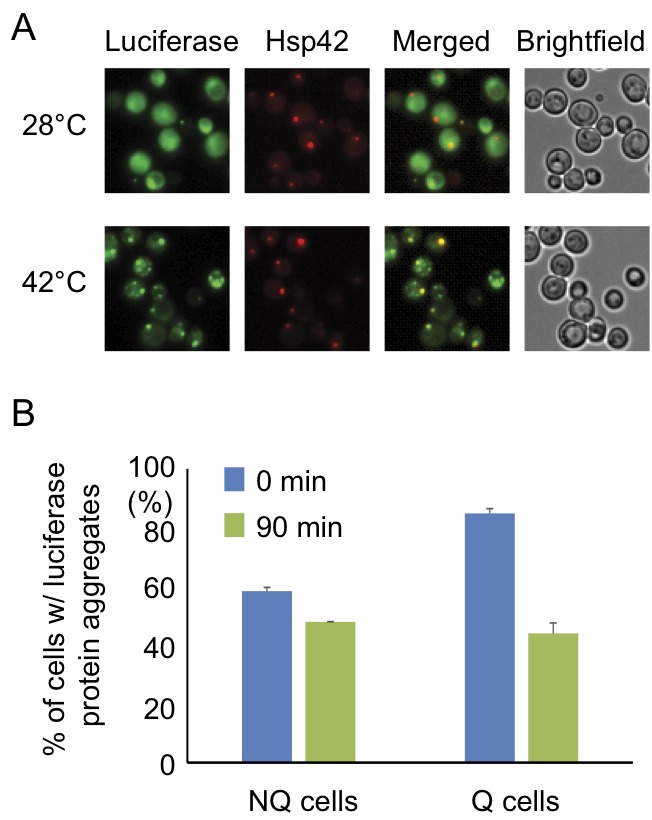
FIGURE 3: Hsp42-SPGs colocalize with heat shock-induced misfolded protein. **(A)** Heat shock-induced misfolded protein colocalizes to Hsp42-SPGs. Three-day stationary phase cells carrying Hsp42-BFP (Red) and an unstable mutant form of GFP-tagged luciferase (Green) were imaged before (28°C) and after (42°C) 30 min of mild heat shock at 42°C. The percentages of Hsp42-SPG-positive cells that colocalized with luciferase dots increased from 16 ± 2% to 70 ± 4% after heat shock. **(B)** Heat shock-induced protein aggregates are cleared more efficiently in Q cells. The luciferase-containing cells were monitored under a time-lapse microscope initiated before heat shock (see Materials and Methods). The numbers of cells containing heat shock-induced protein aggregates were counted immediately after heat shock (0 min) or after recovering at 28°C for 90 min (90 min). 48% of luciferase aggregates ([85 – 44]/85 = 48%) were cleared in Q cells, but only 17% ([58 – 48]/58 = 17%) were cleared in NQ cells. Three samples were analyzed in each condition and at least 100 cells were counted in each sample.

The observation that the Hsp42-SPGs exhibited a different distribution pattern from other granule structures prompted us to further investigate their role in stationary phase cells. One of the important functions of heat shock proteins is to process misfolded proteins induced by environmental stress. We used a mutant construct of a luciferase-GFP fusion protein to test this possibility. This luciferase contains mutations that will cause protein misfolding even under mild heat shock 20. Under normal conditions (28°C), only a small proportion of stationary phase cells (16 ± 2%) displayed one or two weak luciferase aggregates, and most cells had luciferase evenly distributed throughout the cytoplasm. However, after the cells experienced 30 min of 42°C heat shock, a lot of mutant luciferase was misfolded and started to form large aggregates. These luciferase aggregates were observed to colocalize with the Hsp42-SPG dots in 70 ± 4% of Hsp42-SPG-containing cells (Figure 3A), suggesting that one of the Hsp42-SPG’s functions may be as a control center for dealing with misfolded proteins.

Since Hsp42-SPGs showed a higher frequency and brighter intensity in Q cells, we wondered whether Q cells could process the misfolded protein more efficiently than NQ cells. Q and NQ cells were separately subjected to mild heat shock (42°C for 90 min) to induce luciferase misfolding and then clearance of luciferase aggregates was measured. After recovery at 28°C for 90 min, we found that 48% of Q cells had cleared the luciferase aggregates, but only 17% of NQ cells had done so (Figure 3B). These data further support the idea that Hsp42-SPGs may contribute to the stress resistance of stationary phase cultures, especially in Q cells.

### Quiescent cells reenter the mitotic cycle more quickly than non-quiescent cells.

**Figure 4:**
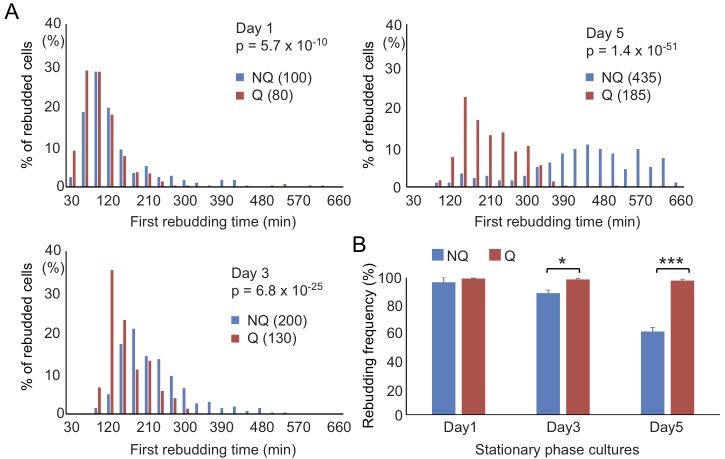
FIGURE 4: NQ cells have lower recovery speeds and viability than Q cells. **(A)** NQ cells take more time to return to the cell cycle than Q cells. Cells growing in YPD for different days were supplied with fresh medium and then time-lapse images were acquired at 10-minute intervals continuously for 10 hours. The first rebudding time of each cell was recorded and the distribution is shown. Numbers in parentheses are the median values of the first budding time (min) in Q and NQ populations. At least 200 cells were counted in each sample, except for the 5-day NQ cells (n = 182). p values were calculated using the Mann-Whitney U test. **(B)** NQ cells quickly lose their ability to rebud. The proportions of Q and NQ cells that rebudded within 10 hours are shown. It has been shown previously that most cells permanently lose their reproductive ability if they do not rebud within 10 hours after the addition of fresh media 19. Three repeat experiments were performed. p values were calculated using the two-tailed Student’s t-test. *: p < 0.05; ***: p < 0.005. Error bars represent the standard error.

NQ cells have been shown to lose their reproductive capability rapidly. However, it has also been suggested that NQ cells continue to divide after diauxic shift 10. To determine whether NQ cells can respond to environmental changes and revert to mitosis more quickly than Q cells, a time-lapse rebudding assay was conducted to determine the time required for each individual cell to reenter the cell cycle after nutrient addition (see Materials and Methods). For Q and NQ cells isolated from 1- or 3-day cultures, there were only small differences in the rebudding time between these two types of cells (median values for rebudding time for 1-day Q, 1-day NQ, 3-day Q, and 3-day NQ cells were 80, 100, 130 and 200 min, respectively). However, in 5-day cultures, NQ cells took a much longer time to rebud than Q cells (the median values for 5-day NQ and Q cells were 435 and 185 min, respectively, Figure 4A). Although many NQ cells already contained a small bud in stationary phase cultures, most of these small buds did not grow immediately after nutrients were added. Consistent with previous observations, NQ cells also exhibited a lower rebudding frequency (61 ± 2.9% of NQ cells vs 98 ± 1.1% of Q cells in 5-day cultures, Figure 4B), indicating that some of them had lost the ability to reproduce. These results indicate that NQ cells not only lose the ability to reenter mitosis during the stationary phase, but they also become less efficient at cell cycle reentry upon fresh nutrient supplementation.

**Figure 5:**
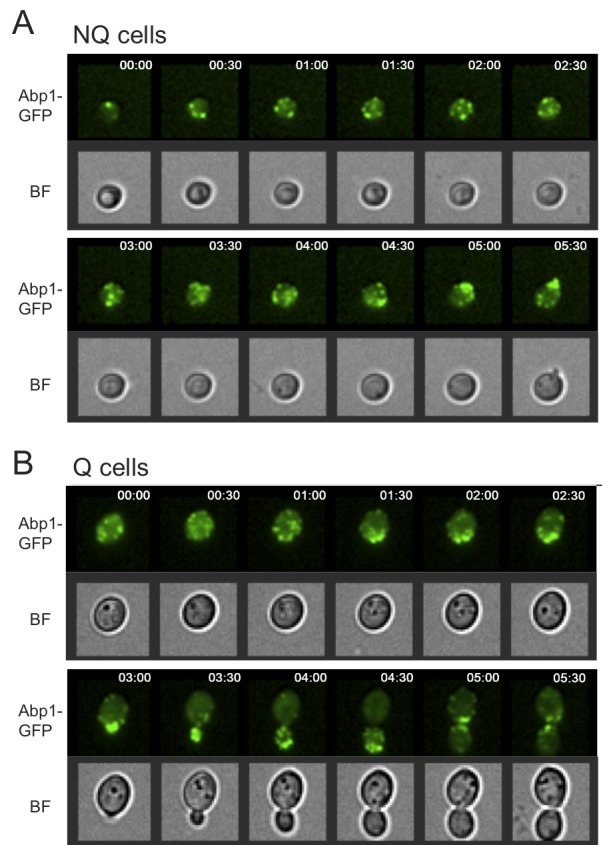
FIGURE 5: Cells reorganize actin bodies before rebudding. Time lapse images of NQ **(A)** and Q **(B)** cells during the rebudding assay. Abp1-GFP was used to monitor the actin structure. In all cells examined, actin bodies were reorganized before the first small bud appeared. The numbers shown in the upper right corner indicate time after addition of fresh media.

Interestingly, in our rebudding assays we also observed that the actin bodies always reorganized to release their components before the cell started to rebud upon the addition of fresh medium (Figure 5). This delay may explain why NQ cells took a longer time than Q cells to reenter mitosis since actin did not form granules in Q cells.

### The cell fate of non-quiescent cells in stationary phase cultures is deterministic. 

**Figure 6:**
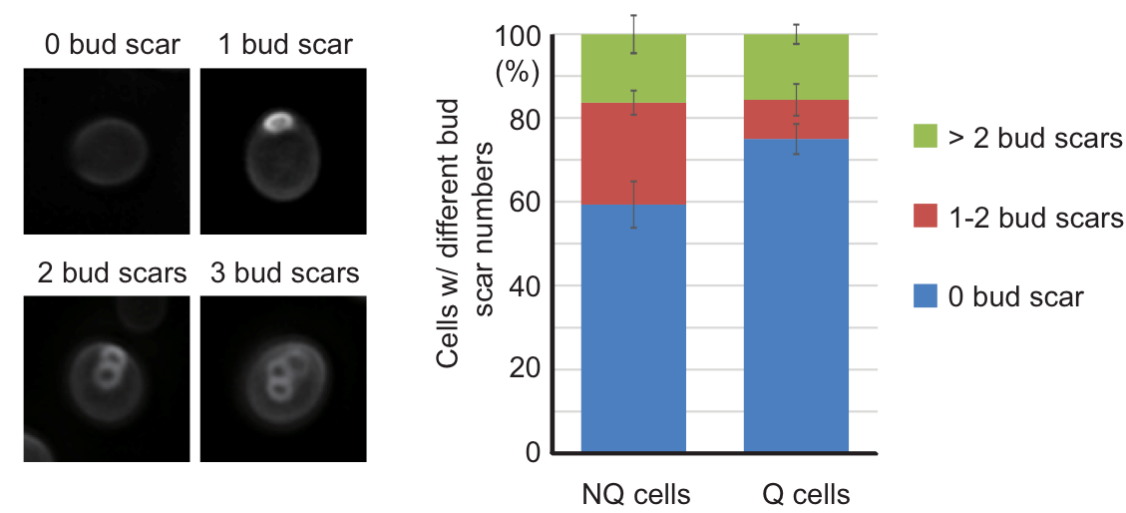
FIGURE 6: A large proportion of NQ cells have one or no bud scar. After the bud scars of Q and NQ cells were stained with calcofluor white, z-stack images were taken and analyzed manually. Three repeats were performed for each cell type and at least 100 cells were analyzed in each repeat. Error bars represent the standard error. The left panel shows typical images of cells with 0, 1, 2 and 3 bud scars.

It has been shown that NQ cells in stationary phase cultures are enriched with cells with more bud scars (representing older replicative ages). However, about 50% of NQ cells did not have any bud scar and 29% of them only had one bud scar 10, indicating that replicative age is not the only factor determining cell fates in stationary phase cultures. Since previous measurements of bud scar numbers were based on the fluorescence intensity detected by flow cytometry 10, we decided to re-examine the bud scar numbers of Q and NQ cells using high-resolution images. Our results were consistent with previous observations that a large proportion of NQ cells (59 ± 5.5%) did not have any bud scar (Figure 6). This result raises the questions whether cell fate is determined stochastically (at least in those NQ cells with one or no bud scar) and whether cell type determination is reversible if stationary phase cells are allowed to reenter mitosis for a few generations before encountering another round of nutrient starvation.

**Figure 7:**
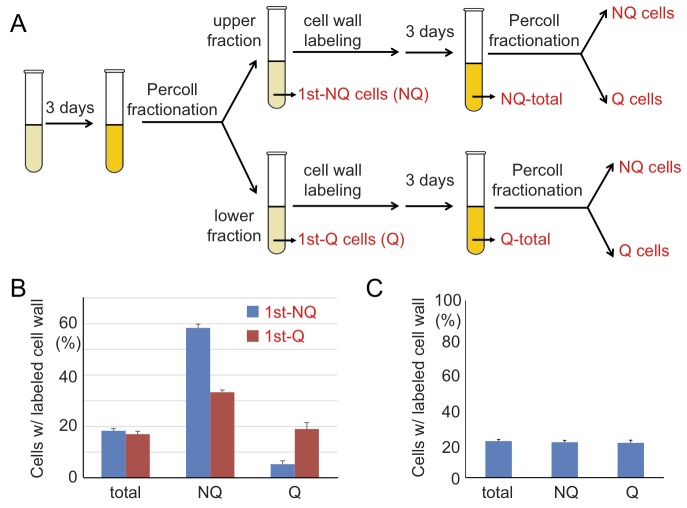
FIGURE 7: The cell fate of NQ cells is mostly irreversible. **(A)** A schematic diagram of the regrowth experiment. Q and NQ cells from 3-day stationary phase cultures were labeled with NHS-Rhodamine and then allowed to separately regrow for another 3 days. The regrown cultures were fractionated again and the cells were analyzed. **(B)** Most NQ cells from the first fractionation are distributed to the NQ (upper) fractions even after regrowth. The mean percentages of cells with Rhodamine-stained cell walls in total stationary phase cultures (total) and the gradient-isolated fractions (NQ and Q) are shown. **(C)** Rhodamine staining does not influence cell fate determination. Cells from stationary phase cultures were stained with NHS-Rhodamine and then mixed with unstained cells from the same culture. The mixed cell cultures were fractionated and the percentages of Rhodamine-stained cells in different fractions were analyzed. In all regrowth experiments, at least 3 repeat experiments were performed and at least 100 cells were counted in each repeat. Error bars represent the standard error.

It is known that during cell divisions, yeast mother cells keep the old cell wall and the proteins on it. Once the cell wall is stained with fluorescent dye, the mother cells will remain fluorescent even after several generations, but the newly born daughter cells will not. To further investigate how cell fates are determined, we used cell wall labeling to mark the original populations and then tracked their cell fates in later generations. Q and NQ cells were first labeled with a fluorescent dye (Rhodamine) and diluted in fresh medium to grow for a few generations before entering another round of stationary phase. Next, the stationary phase cultures were fractionated again and the proportions of labeled cells were counted in each fraction (see Materials and Methods, Figure 7A). We found that a proportion of labeled Q cells were redistributed to the portion of NQ cells in the second fractionation. More strikingly, a majority of labeled NQ cells stayed in the NQ fraction despite having reentered mitosis for a few cell divisions (Figure 7B). When the unlabeled daughter cells were analyzed, we observed that daughter cells derived from labeled NQ cells were more likely to become NQ cells too (Figure S2).

To rule out the possibility that the distribution bias was simply due to cell wall labeling, cell wall-labeled cells were mixed with unlabeled cells from the same stationary phase culture and then the mixed cells were fractionated to measure their distributions. Similar frequencies of labeled cells were observed in both Q and NQ fractions, indicating that cell wall labeling does not result in a biased distribution during cell fractionation (Figure 7C). Our data suggest that once cells are committed to becoming NQ cells, their cell fate is mostly fixed and reentry into mitosis will not reverse it. In contrast, Q cells are more flexible and have the ability to become Q or NQ cells after reverting back to mitosis.

## DISCUSSION 

Our current study reveals that previously identified granule-like structures form differently in Q and NQ cells isolated from stationary phase yeast cultures. Surprisingly, most of these structures, including actin bodies, PSGs, stress granules and P-bodies, are highly enriched in the NQ cell fraction. Only Hsp42-SPGs preferentially form in Q cells 9. The biased distribution suggests that the formation of these granule structures strongly correlates with the physiological state of stationary phase cells. The NQ cell fraction has been suggested to comprise more senescent cells that exhibit high levels of reactive oxygen species and quickly lose their reproductive ability during the stationary phase 10. Our results also demonstrate that NQ cells respond to fresh medium more slowly, probably due to the time required for them to reorganize granule structures. Why should NQ cells develop these specific granule structures if they are not as vital to Q cells in the same culture? One possible explanation is that the formation of granules is a protective mechanism to isolate critical proteins or RNA from the harmful cytosolic environment whenever homeostasis of a cell is severely perturbed. Under this hypothesis, granule formation represents an ultimate strategy for NQ cells to survive a bit longer. In contrast, Q cells will not form these granules before their physiological state starts deteriorating. In agreement with this idea, more Q cells were observed to form these granules in 2-week cultures in which the colony-forming ability of Q cells had dropped to about 60% (data not shown) 10.

Among the granule structures examined by us, the Hsp42-SPG was the only one found to have an increased frequency and intensity in Q cells, suggesting that Hsp42-SPGs may play a more active role at the early stage of stationary phase cells. In log phase cells, Hsp42 positively regulates the formation of protein foci containing misfolded proteins upon heat stress and the absence of Hsp42 reduces cell growth rate at high temperature 21,22. During replicative aging, Hsp42 is also required for the formation of age-associated protein deposits and influences the aging process of mother cells 23. It has previously been shown that Hsp42-SPGs contain the small heat shock proteins Hsp26 and Hsp42, and a metacaspase-like cysteine protease Mca1 (alias as Yca1) that is involved in clearance of insoluble protein aggregates 19,24. Upon entry into the stationary phase, expression levels of many heat shock proteins are induced and the stress-responsive transcriptional factors Msn2/Msn4 are important for quiescence entry 25,26. Hsp42-SPGs probably coordinate different molecular chaperones and proteases to work as a protein quality control center that confers tolerance to various stresses in Q cells. This hypothesis is supported by the observation that when Q cells were subjected to heat shock stress, misfolded proteins were localized to Hsp42-SPGs and protein aggregates were cleared more efficiently in Q cells. Therefore, Hsp42-SPGs may protect cells from senescence by actively maintaining protein homeostasis in the Q cell. In contrast, the Hsp42-SPGs observed in NQ cells were much smaller in size and mostly did not contain Hsp26, suggesting that they may have other functions. One possibility is that Hsp42-SPGs in NQ cells simply work as protein storage sites for permanently damaged proteins or critical proteins for mitotic entry. Further investigations will be required to elucidate the detailed differences between these two types of Hsp42-SPGs.

Currently, it remains unclear how cells make the decision to differentiate into one of the two cell types. Davidson and colleagues 11 proposed that the differentiation of Q and NQ cells is determined by an epigenetic change before entering the post-diauxic phase. However, the physiological difference between NQ and Q cells is so large that it becomes extremely challenging to identify the determining mechanism of cell differentiation. Our cell cycle reentry experiments reveal that the cell fate of NQ cells is largely unaltered even if NQ cells are induced to reenter mitosis. Comparing the mitotic populations derived from NQ and Q cells (or virgin log phase cells) provides another approach to screen for cell fate determining factors because the physiological states of these two populations may be much less diverse and the key difference can be pinpointed more easily.

It appears that the development of Q and NQ populations in stationary phase cultures is well programmed. Why should stationary phase cells differentiate into Q and NQ cell types? It has been proposed that differentiation into Q and NQ cells is a risk-spreading, bet-hedging strategy given the fact that Q cells are more resistant to stress and have a longer lifespan, whereas the high mutability of NQ cells increases their chance of adapting to harsh environments 11,27. The other possibility is that differentiation allows the majority of the population to benefit from cooperative behavior between two cell types. Previous studies have revealed that when growing on a solid agar plate, yeast cells inside a colony also differentiate into two cells types (U cells and L cells), with L cells providing nutrients to support U cells 28. Because NQ cells lose reproductive ability quickly, they may simply serve as nutrient reserves for Q cells 11. Alternatively, the NQ cell may be able to secrete or release some microbial inhibitory compounds to protect the population from invasion by other microbes. Stationary phase cultures are most vulnerable to invasion since yeast cells have stopped dividing but many other microorganisms can still grow in this nutrient depleted condition. If NQ cells work to “guard” the population, it explains why NQ cells lose their reproductive ability in a few days in stationary phase, but still maintain their metabolic viability for several weeks 10. Further investigation of the physiology of NQ cells and the lifespans of Q cells in isolated or mixed populations will help us to understand the real function of cell differentiation in stationary phase cultures.

Stationary phase cultures represent a complicated cell population comprising at least two different cell types. Our data indicate that Q and NQ cells are not only different in their physiological states, but also form different cytosolic structures. Some granule structures have been suggested to play important roles in regulating gene expression or protein functions. Understanding how these structures develop and function will provide insight into the mechanisms of chronological aging and cell differentiation.

## Materials and Methods

### Yeast strains and growth conditions

All yeast strains used in this study were derived from the S288C strain background. Yeast strains containing the C-terminally GFP-tagged proteins were obtained from the chromosomal GFP-tagged yeast collection 29. The GFP and yomTagBFP2 tags were inserted in-frame at the C-terminus of the coding region of a gene, as described previously 30,31. All fusion proteins were expressed under their endogenous promoters. Cells were cultured at 28°C with aeration in liquid YPD medium before being examined by microscopy. Distilled water was added regularly into the liquid culture to compensate for water loss from evaporation.

### Cell fractionation

Yeast cells were fractionated following the protocol described previously with some modifications 10. Percoll (GE Healthcare, Little Chalfont, Buckinghamshire, United Kingdom) and 1.5 M NaCl were mixed in a 9:1 ratio, and the mixture was further diluted with 0.15 M NaCl in a 9:1 ratio. 1.8 ml diluted Percoll mixture was added into a 2 ml eppendorf tube and centrifuged at 21130 x g for 15 min at 4°C to form the gradient. Cells from 500 μl stationary phase cultures were harvested, re-suspended in 100 μl 0.05 M Tris buffer, and overlaid upon the Percoll mixture. The cells were fractionated by centrifugation at 400 x g for 1 hr. Q and NQ cells were collected with pipettes and washed before examinations.

Under bright field microscopy, Q and NQ cells displayed different cell morphologies (Figure S3), which were consistent with previous observations 10. In particular, the vacuoles in NQ cells were larger and more obvious compared to those in Q cells. By examining vacuole morphology, Q and NQ cells could be easily distinguished in unfractionated stationary phase cell cultures. The ratios of Q and NQ cells in 3- and 5 - day cultures were close to 85:15 and 65:35, respectively (Figure S3). In our rebudding assay, we found that the particular morphology of vacuoles in NQ cells disappeared and became similar to that of Q cells once fresh nutrients were available (Figure S4).

### Monitoring of protein localization

Yeast cells were diluted with PBS and loaded into a Glass Bottom ViewPlate®-96 F (PerkinElmer, Waltham, MA) coated with concanavalin A (C2010, Sigma-Aldrich, St. Louis, MO). Then the plates were centrifuged to attach cells to the glass bottom and the images were obtained using the ImageXpress Micro XL system (Molecular Device, Sunnyvale, CA). All the images were analyzed manually using ImageJ (http://rsbweb.nih.gov/ij ).

### Monitoring of misfolded protein localization and clearance of misfolded protein aggregates

To monitor misfolded protein in the cells, the double-mutant construct of luciferase-EGFP 20 was cloned to the pCM189 plasmid 32. The cells carrying the plasmid were cultured in CSM-URA media for 3 days and then monitored using the ImageXpress Micro XL system. In order to induce misfolding of the luciferase-EGFP mutant protein, the heater of the ImageXpress Micro XL system was set to start from 42°C at first and time-lapse images were obtained every 15 minutes. After 90 minutes of heat shock, the temperature was changed to 28°C for monitoring the clearance of misfolded protein aggregates. In order to monitor the clearance of heat shock-induced misfolded protein, only cells that did not contain any luciferase aggregates before the heat shock were counted in the clearance experiment. The cells were classified as cleared when all luciferase aggregates inside the cells disappeared and became diffused in cytoplasm.

### Rebud assay and survival rate measurement

YPD agarose (2%) pads were spliced to the size of 4.5 x 4.5 mm. Fractionated cells were loaded onto the surface of spliced YPD agarose pads. After 3-5 min of air-drying at room temperature, the pads with cells were turned upside down and put into the wells of a Glass Bottom ViewPlate®-96 F. Time-lapse images were acquired using the ImageXpress Micro XL system every 10 minutes and analyzed manually using ImageJ. In the case of budded cells found at the beginning of the assay, the first rebudding time was counted when the bud started to enlarge. For single cells found at the beginning of the assay, the first rebudding time was counted when a small bud started to emerge. Cells that did not rebud within 10 hours were classified as non-reproductive cells, and the rebudding frequencies were calculated.

### Yeast cell staining

Fractionated stationary phase cells were pelleted and washed once with PBS before dye staining. For bud scar staining, cells were resuspended in 200 μl of 0.1 mg/ml calcofluor white (Fluorescent Brightener 28) in distilled water for 5 min. For cell wall staining, cells were resuspended in 500 μl of 25 μg/ml NHS-Rhodamine in PBS with 0.1 M NaHCO_3_ for 3 min. 500 μl exhausted YPD medium was added to stop staining and the cells were washed three times with PBS.

### Labeling and regrowth of Q and NQ cells

Yeast cells were grown in YPD for 3 days and 100 ml of the cultures were collected and fractionated. Q and NQ cells were labeled with NHS-Rhodamine and then separately re-suspended in 2.25 ml filtered exhausted YPD at a cell density similar to that of 3-day YPD cultures. 0.75 ml fresh YPD was added into the cell suspension and the cells were regrown for 3 days before another round of cell fractionation. The fluorescent images of fractionated Q and NQ cells were analyzed to calculate the percentage of cells with cell wall labeling. 3-day stationary phase cultures were used in this experiment because most 3-day NQ cells still have rebudding abilities similar to Q cells (Figure 4B).

## SUPPLEMENTAL MATERIAL

Click here for supplemental data file.

All supplemental data for this article are available online at http://microbialcell.com/researcharticles/differentiated-cytoplasmic-granule-formation-in-quiescent-and-non-quiescent-cells-upon-chronological-aging/
