# Supplementary material for: Differentiated cytoplasmic granule formation in quiescent and non-quiescent cells upon chronological aging
Source: Microb Cell. 2016 Mar 3;3(3):109–19. doi: 10.15698/mic2016.03.484 (PMC5349021; doi:10.15698/mic2016.03.484)
Supplement: Supplementary file 1 [file mic-03-109-s01.pdf]

Figure S1

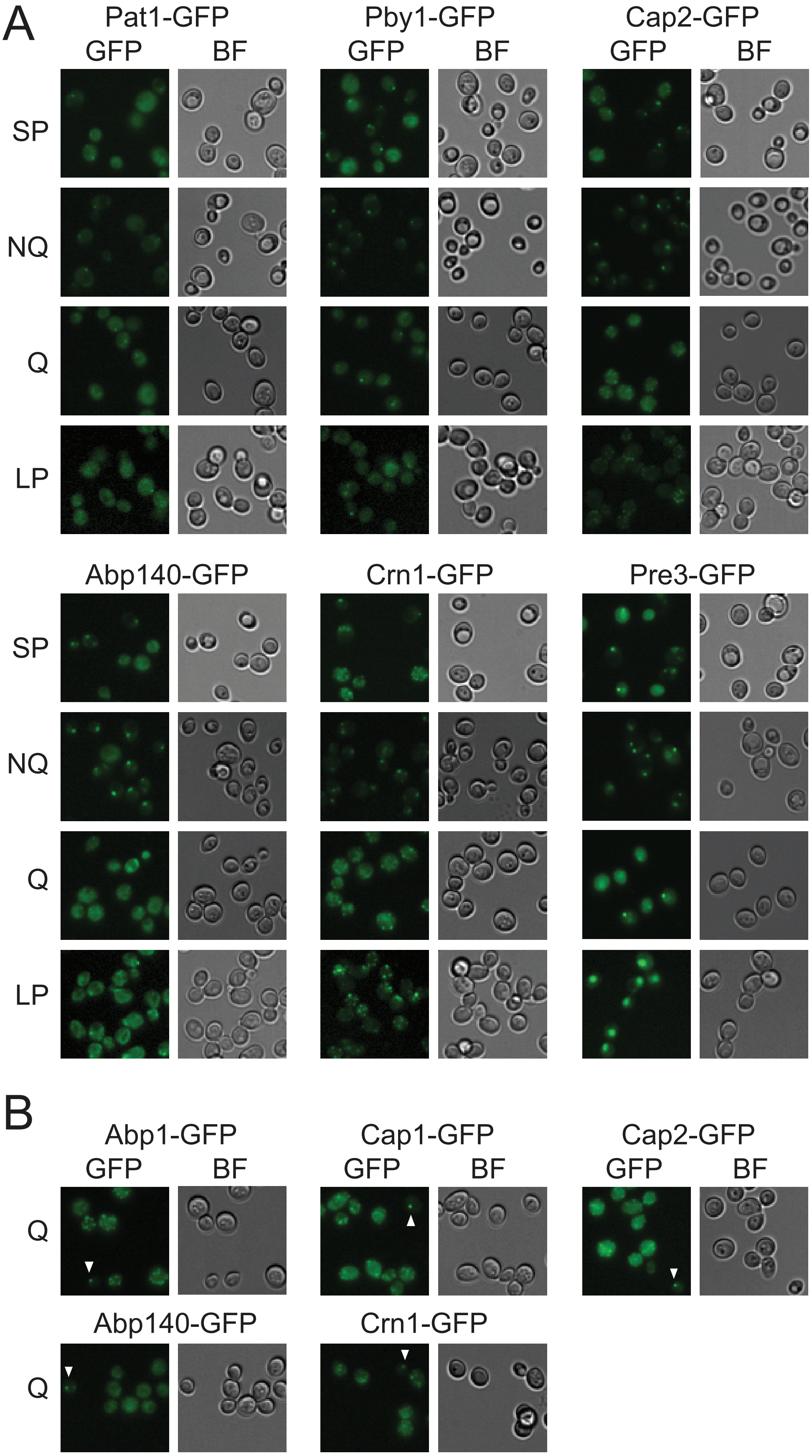

Figure S1. Representative images of stationary phase-specific granules in Q and NQ cells.
(A) Differential formation of stationary phase-specific granules in Q and NQ cells. Components of the P-body, actin body and proteasome storage granule that are listed in Table 1 but not shown in Figure 1. The fluorescence images of total stationary phase cultures (SP) and the fractionated cells (Q and NQ) from the same strain are shown under identical brightness and contrast settings. Log-phase (LP) images were taken from exponential cell cultures whose OD600 values were between 0.4 and 0.6. BF: bright field. (B) Representative images of the actin body in Q cells. Cells with the actin body displayed a single round dot in the cytosol (indicated by the arrowhead). Majority of the Q cells did not contain the actin body and exhibited multiple dots/punctates which were similar to the localization patterns observed in log-phase cells.

Figure S2

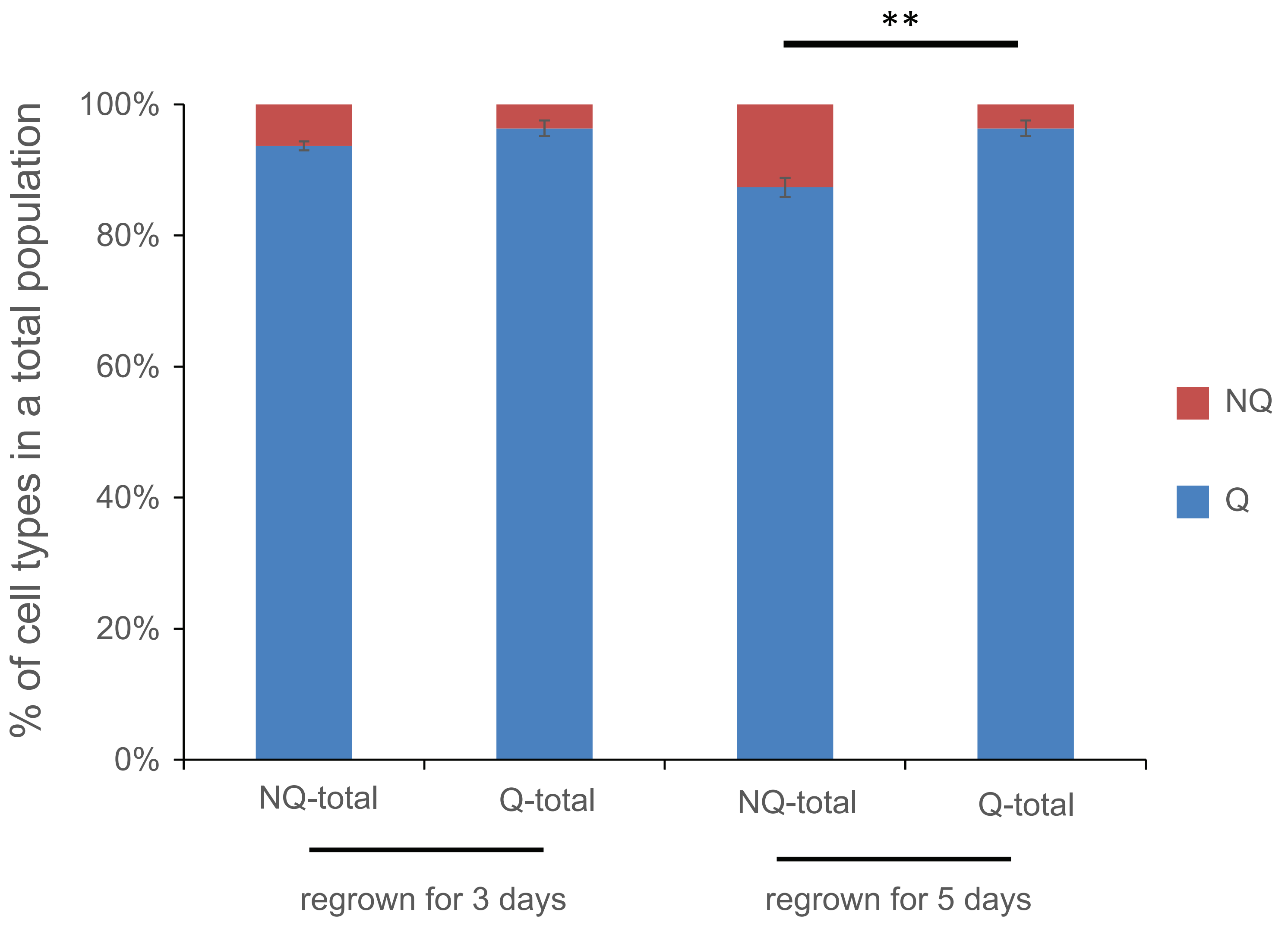

Figure S2. Daughter cells derived from NQ parents are biased toward the NQ differentiation.

Q and NQ cells from 3-day stationary phase cultures were labeled and regrown in YPD for 3 or 5 days and unlabeled daughter cells in the NQ-total and Q-total populations (see also Figure 7A) were analyzed. The proportions of Q and NQ daughter cells were measured based on the vacuole morphology (see also Figure S3). The percentages of NQ cells were compared using a two-tailed t-test. **: p-value < 0.01.

Figure S3

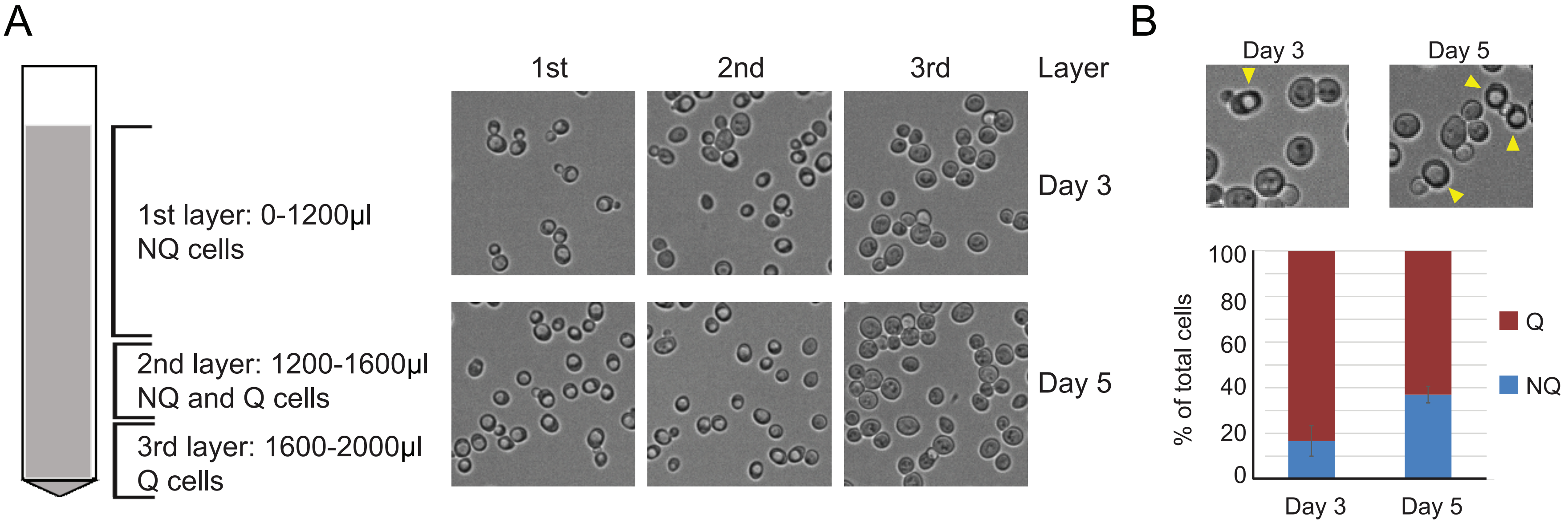

Figure S3. The proportions of Q and NQ cells in 3- or 5-day cell cultures.
(A) Vacuole morphology correlates well with the cell type of stationary phase cells. After the stationary phase cells were fractionated using the Percoll gradient, the first layer of the gradient mainly contained NQ cells and the third layer contained Q cells. Cells in the second layer were a mixture of NQ and Q cells. Bright field images showed that NQ cells in the first layer carried large vacuoles, while Q cells in the third layer did not. These results are consistent with the previous observation [10]. (B) The proportions of Q and NQ cells in 3- and 5-day cultures. The upper panel shows typical cell images in 3- and 5-day cultures. The yellow arrowheads indicate NQ cells with large vacuoles. The vacuole morphology was used to distinguish Q and NQ cells in the stationary phase population. The ratios of Q and NQ cells in 3- and 5-day cultures are 85:15 and 65:35, respectively.

Figure S4

NQ cells

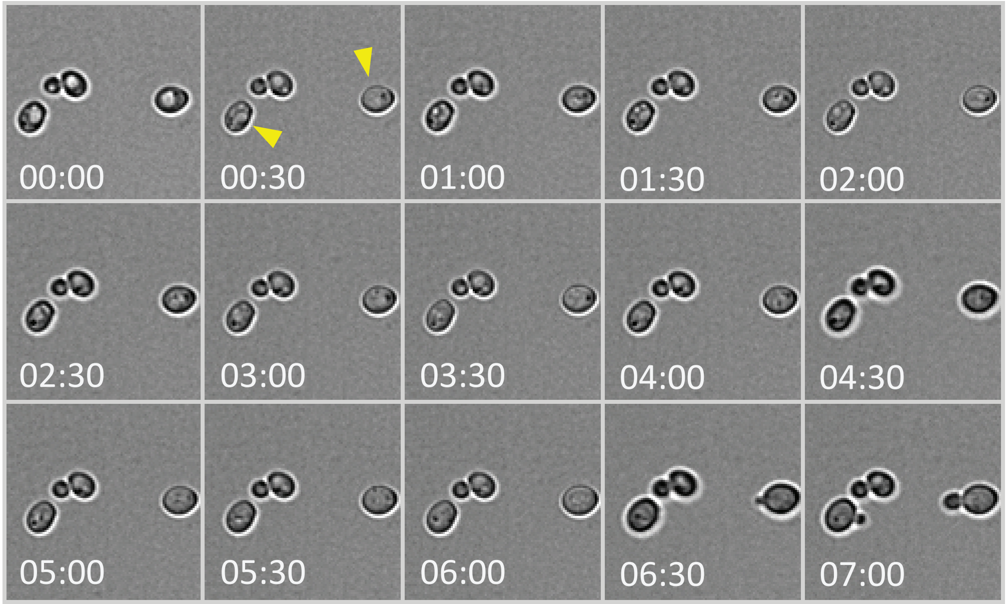

Q cells

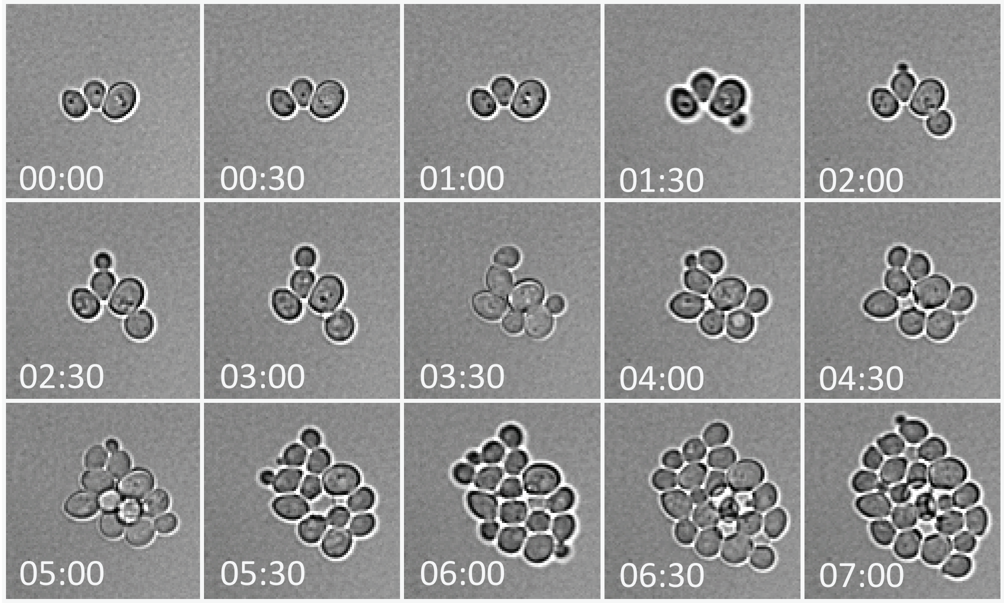

Figure S4. The special morphology of vacuoles in NQ cells disappears during rebudding. Time lapse images of NQ (A) and Q (B) cells during the rebudding assay. The yellow arrowhead indicates the vacuole of NQ cells. The numbers shown in the lower left corner indicate time after addition of fresh media.
